# PPR596 Is Required for *nad2* Intron Splicing and Complex I Biogenesis in Arabidopsis

**DOI:** 10.3390/ijms25063542

**Published:** 2024-03-21

**Authors:** Aqib Sayyed, Baoyin Chen, Yong Wang, Shi-Kai Cao, Bao-Cai Tan

**Affiliations:** Key Laboratory of Plant Development and Environmental Adaptation Biology, Ministry of Education, School of Life Sciences, Shandong University, Qingdao 266237, China; aqib.sayyed@yahoo.com (A.S.); ba_oyin_love@126.com (B.C.); wangy07@sdu.edu.cn (Y.W.); caoshk5233@163.com (S.-K.C.)

**Keywords:** PPR596, C-to-U RNA editing, *nad2* intron splicing, complex I assembly, mitochondrial respiratory chain, Arabidopsis

## Abstract

Mitochondria are essential organelles that generate energy via oxidative phosphorylation. Plant mitochondrial genome encodes some of the respiratory complex subunits, and these transcripts require accurate processing, including C-to-U RNA editing and intron splicing. Pentatricopeptide repeats (PPR) proteins are involved in various organellar RNA processing events. PPR596, a P-type PPR protein, was previously identified to function in the C-to-U editing of mitochondrial *rps3* transcripts in Arabidopsis. Here, we demonstrate that PPR596 functions in the cis-splicing of *nad2* intron 3 in mitochondria. Loss of the PPR596 function affects the editing at rps3eU1344SS, impairs *nad2* intron 3 splicing and reduces the mitochondrial complex I’s assembly and activity, while inducing alternative oxidase (AOX) gene expression. This defect in *nad2* intron splicing provides a plausible explanation for the slow growth of the *ppr595* mutants. Although a few P-type PPR proteins are involved in RNA C-to-U editing, our results suggest that the primary function of PPR596 is intron splicing.

## 1. Introduction

Mitochondria are the major cellular organelles important for energy production and the synthesis of essential metabolites. Mitochondria evolved 1.5 billion years ago via the symbiosis of an early anaerobic eukaryote cell engulfing an α-protobacterium. During evolution, most mitochondrial genes either shifted to the nucleus or were lost [1]. Animal mitochondrial genomes are concise, with 37 genes encoding merely 13 proteins, 22 tRNAs, and 2 rRNAs [2]. Conversely, plant mitochondrial genomes, notably larger and structurally diverse, contain around 60 genes that primarily code for essential components like rRNAs, tRNAs, and subunits of respiratory complexes (CI to CV). Despite their compact size, mitochondria require numerous nuclear-encoded proteins for DNA maintenance, transcription, translation, and post-transcriptional processes like RNA editing and intron splicing [3,4].

Intron splicing is an essential step of post-transcriptional processing, and plants use post-transcriptional processes to regulate mitochondrial gene expression [5]. Many mitochondrial genes contain introns that must be removed and neighboring exons joined to code for functional proteins. *Arabidopsis thaliana* mitochondria have been found to harbor twenty-three introns, including four in NADH dehydrogenase subunit1 (*nad1*), *nad2*, *nad5*, and *nad7*, three in *nad4*, and one in each of the cytochrome c oxidase subunit2 (*cox2*), cytochrome c maturation subunit F C-terminus (*ccmFc*), ribosomal protein large subunit2 (*rpl2*), and ribosomal protein small subunit3 (*rps3*). Plant mitochondrial genomes contain *cis-* and *trans*-introns. Thus, the *trans*-introns in *nad1*, *nad2*, and *nad5* transcripts require *trans*-splicing [6]. Regardless of intron configuration, introns are classified into group I and group II based on their structure and splicing mechanism. Group I introns comprise 10 conserved paired regions, P1–P10, in the core secondary structure. The splicing of group I introns is facilitated by two-step phosphoryl transfer reactions that use exogenous guanosine as a cofactor [7,8]. Only one group I intron has been discovered in the cox1 gene of multiple plant species [9]. Almost all mitochondrial introns are group II introns. Group II introns are characterized by six stem-loop structures (e.g., domains I-VI), and the splicing mechanism resembles that of nuclear pre-mRNA splicing, involving two transesterification steps and the intron being released as a lariat [10]. In bacteria, the group II intron domain IV encodes a maturase, which is crucial for facilitating the splicing of its cognate group II intron. In higher plants, only one maturase, MatR encoded by nad1 intron 4, is found in the mitochondrial genome [11]. Meanwhile, four maturases, named nMAT1 to nMAT4, are encoded by the nuclear genome in Arabidopsis. Higher plants such as angiosperms, however, have evolved numerous nucleus-encoded factors to facilitate intron splicing, including CRM (Chloroplast RNA splicing, ribosome maturation (CRM), Plant Organellar RNA Recognition (PORR) domain protein, Regulator of Chromosome Condensation (RCC), and pentatricopeptide repeat (PPR) protein [12].

PPR proteins are a prominent group of proteins in land plants, with over 450 members in *Arabidopsis thaliana* predominantly localized in chloroplasts or mitochondria [13,14]. Typically characterized by 2–30 tandem repeats of a 35-amino-acid motif, each PPR motif folds into a pair of antiparallel helices [15,16]. They are classified into the P and PLS subfamilies based on motif structures, with the latter further divided into PLS, E, and DYW subclasses based on the presence of E or DYW motifs in the C-terminal sequences [17]. Members of the P subfamily participate in various organellar post-transcriptional processes, including RNA stabilization and splicing, whereas PLS subfamily proteins are involved in RNA editing [16,18]. PPR proteins bind to their cognate nucleotides in a target-specific manner. Each PPR repeat of a PPR protein binds to a single RNA nucleotide in a modular pattern. The amino acids at the fifth and thirty-fifth positions determine the specificity of the PPR protein [16,19,20,21]. PPR proteins play a critical role in mitochondrial intron splicing, and mutations in the genes encoding these proteins lead to defects in splicing, impacting mitochondrial respiratory complex assembly and causing growth and developmental abnormalities [22,23,24,25,26,27]. Various PPR proteins contribute to the splicing of introns in multiple genes, underscoring their significance in organelle biogenesis [23,28].

PPR596 encodes a mitochondrion-localized P-type PPR protein. It was previously reported to be involved in RNA C-to-U editing at the rps3eU1344SS site of the *rps3* transcript in *Arabidopsis thaliana* [29]. In this study, we identified that PPR596 functions in intron splicing, probably the primary function that contributed to the observed slow growth phenotype in its mutants. The loss of PPR596 function results in defective *cis*-splicing of *nad2* intron 3, leading to a significant reduction in the assembly and activity of complex I, increases the expression of alternative oxidase genes, and ultimately results in the slow growth phenotype in *Arabidopsis thaliana*.

## 2. Results

### 2.1. PPR596 Encodes a Mitochondrion-Localized PPR Protein

**Figure 1:**
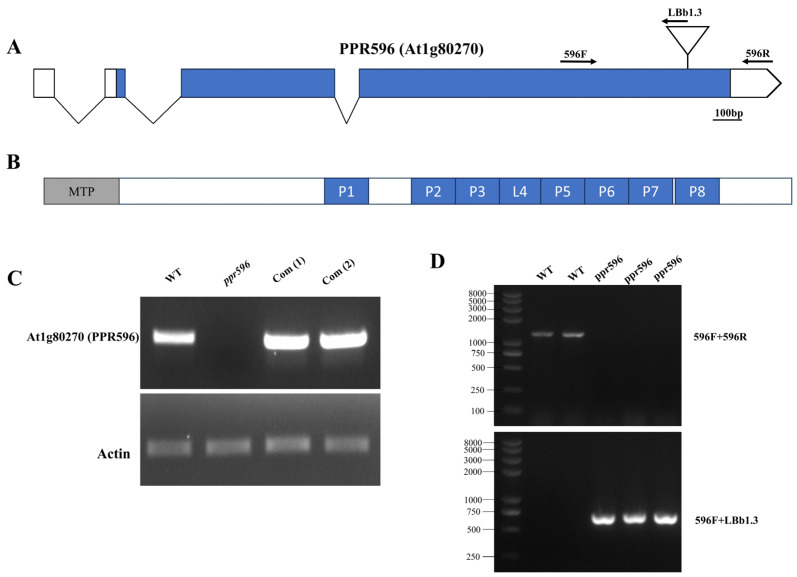
Gene topology, characterization, and complementation of the homozygous SAIL_367_A06 (*ppr596*) mutant. (**A**) Gene structure showing introns and exons. Blue indicates the coding region (exon), white indicates the untranslated regions, and oblique lines in the structure represent introns of the gene. The triangle indicates the T-DNA insertion site. The T-DNA insertion site in the coding region of SAIL_367_A06 (*ppr596*) is located at 1584bp from the start codon and 207bp upstream of the stop codon. Arrows indicate the positions of the primers used for genotyping. (**B**) Schematic representation of the PPR596 protein, showing mitochondrial targeting peptide (MTP) in a gray box, and blue boxes represent PPR domains in the protein. (**C**) Expression analysis of *PPR596* in WT, *ppr596* mutant, and complementation lines by RT-PCR. Actin expression was used as a loading control. (**D**) Genotyping of the plants for identifying homozygous *ppr596* mutant plants using primers indicated in the gene structure.

AT1G80270 encodes a mitochondrion-localized P-type PPR protein consisting of 596 amino acids and was previously named PPR596 [29]. Unlike most of the PPR genes that are intronless, *PPR596* contains three introns, with two introns in the coding region and one in the 5′-untranslated region (Figure 1A). This PPR protein contains eight P-type PPR repeats, according to the PPRCODE prediction software (http://yinlab.hzau.edu.cn/pprcode) (accessed on 20 August 2023) (Figure 1B).

TargetP [30], MitoFates (aist.go.jp) (accessed on 21 April 2023), and ExPASy portal (https://www.expasy.org) (accessed on 21 April 2023) collectively indicate the presence of 61 amino acid mitochondrial targeting sequences at the N-terminal region of PPR596 (Figure S1). Experimental evidence confirmed the mitochondria localization of PPR596 [29]. Additionally, the in silico 3D structure prediction results obtained from the AlphaFold server [31] suggest that PPR596 has a typical PPR helical fold (Figure S2), including an inner basic core that serves as the RNA-binding surface. This finding is consistent with previous studies on plant PPR10 proteins [32].

### 2.2. Characterization of the ppr596 Mutants

The T-DNA insertion line (SAIL_367_A06) was obtained from the Arabidopsis Biological Resource Center (ABRC; Columbus, OH, USA). Homozygous plants were identified by PCR analysis (Figure 1D). The T-DNA insertion was confirmed by sequencing and was found to be located 207 nt upstream of the stop codon of *PPR596* (Figure 1A). The expression of wild-type PPR596 was not detected in homozygous mutants, whereas the transcript levels of *Actin2* (At3g18780) were not affected (Figure 1C).

**Figure 2:**
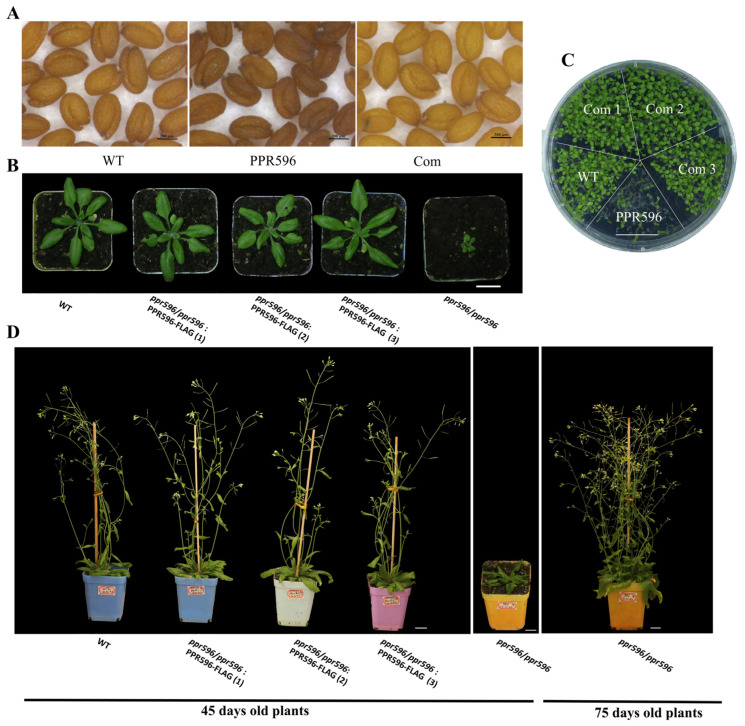
Phenotypic analysis of *ppr596* mutant plants. (**A**) Morphology of WT, *ppr596* homozygous mutant, and complementation line seeds under a light microscope. (**B**) Four-week-old seedlings of the WT, *ppr596* homozygous mutant, and complementation lines (*ppr596*/*ppr596*: PPR596-FLAG) in pots. The four-week-old homozygous *ppr596* mutant (*ppr596*/*ppr596*) was very small compared to WT plants. (**C**) Two-week-old seedlings grown on MS medium. (**D**) 45-day-old plants of WT, *ppr596* homozygous mutant, and complementation lines (*ppr596*/*ppr596*: PPR596-FLAG) in pots. The homozygous *ppr596* mutant exhibited slower growth and delayed maturation compared to both WT and complementation plants (first from the right, 75 days old). The bar in (**B**–**D**) indicates 2 cm.

The *ppr596* mutants displayed a noticeably slower growth phenotype than the wild-type plants. The *ppr596* seed germination was significantly delayed, and after two weeks, the mutant plants were visibly smaller than their wild-type counterparts (Figure 2C). Based on soil-based phenotypic analysis, plant growth was retarded throughout the germination stage. The *ppr596* mutant plants had a delay of approximately 30 days at the onset of flowering compared with the wild-type plants (Figure 2B,D). Under the light microscope, mature seeds of the mutant plants were smaller and wrinkled, unlike mature wild-type seeds (Figure 2A). Mutant plants expressing the open reading frame of AT1G80270 under the control of the 35S promoter and flag-tag at the C terminal rescued the slow growth phenotype and seed development. They grew similarly to wild-type plants, indicating that the lack of PPR596 is the cause of the slow growth phenotype.

### 2.3. Expression of nad2 Is Reduced in ppr596

**Figure 3:**
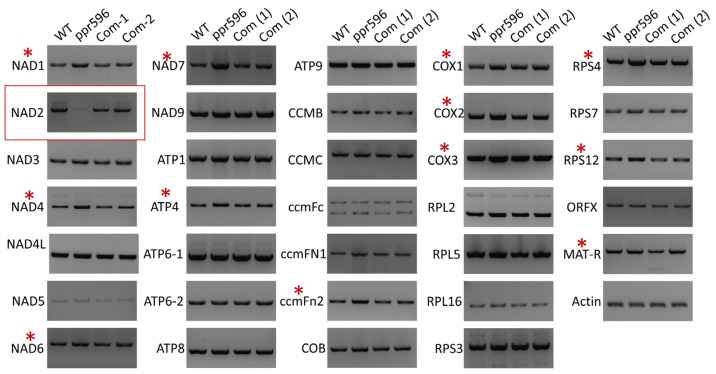
Analysis of mitochondrial gene transcripts in the *ppr596* mutant. RT-PCR analysis of 33 mitochondrial protein-coding genes in the *ppr596* mutant with WT and complemented plants. Actin expression was used as the loading control. Transcripts of *nad2* (highlighted in red box) were severely decreased in the *ppr596* mutant. The expression of some genes increased in the *ppr596* mutant, as indicated by the asterisk.

PPR proteins are RNA-binding proteins involved in RNA metabolism including RNA maturation, splicing, and C-to-U RNA editing in mitochondria and chloroplasts. PPR596 is a P-type PPR protein that has previously been reported to partially decrease the editing of mitochondrial ribosomal protein S3 (RPS3) transcripts [29]. PPR596, POCO1 [33] and PPME [34], have been frequently cited as exceptional P-type PPR proteins for their role in RNA-editing [16,35,36,37]. However, most P-type PPR proteins are primarily associated with RNA stabilization and splicing in organelles. To further investigate the possible functions of PPR596, all mature transcripts of mitochondrial protein-coding genes were examined by semi-quantitative RT-PCR in the *ppr596* mutant, WT, and complementation lines using the primers listed in Table S1. Notably, we observed a significant reduction in the abundance of mature *nad2* transcripts in the *ppr596* mutant compared with the WT and complementation lines (Figure 3). Additionally, the expression levels of various mitochondrial transcripts, including *nad1*, *nad4*, *nad6*, *nad7*, *atp4*, *ccmFn2*, *cox1*, *cox2*, *cox3*, *Rps4*, *rps12*, and *MAT-R*, were higher in the *ppr596* mutant than in WT and complemented plants.

### 2.4. PPR596 Is Required for Splicing of the nad2 Intron 3

**Figure 4:**
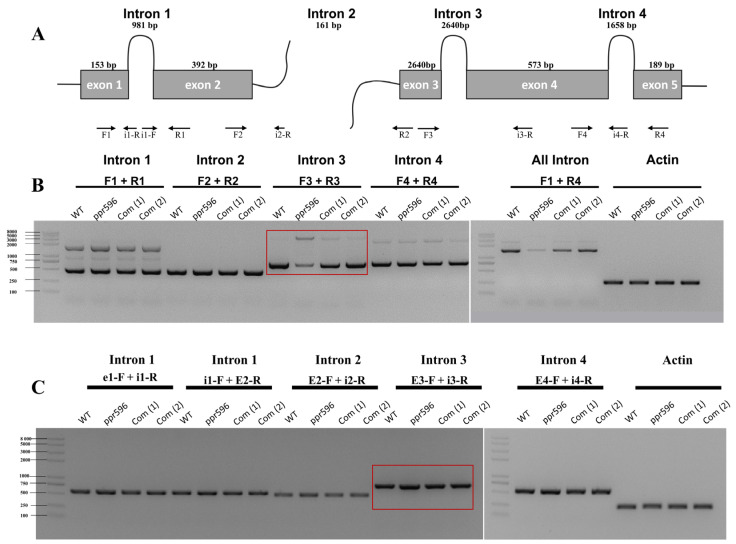
PPR596 is involved in the splicing of *nad2* intron3. Splicing events of *nad2* were examined in WT, *ppr596*, and complementation lines by RT-PCR. Actin expression was used as a loading control. (**A**) The structure of the *nad2* pre-mRNA consists of two precursor RNA transcripts. The first precursor RNA consists of two exons and one intron, while the second consists of three exons and two introns. The maturation of *nad2* mRNA requires proper splicing of three *cis* (introns 1, 3, and 4) and one *trans* (intron 2) intron splicing. Arrows indicate the positions of primers used to detect unspliced introns. (**B**) RT-PCR analysis revealed a significant decrease in the splicing efficiency of *nad2* intron 3 in the *ppr596* mutant, as highlighted by the red box. (**C**) RT-PCR analysis showing the accumulation of unspliced intron 3 in the *nad2* transcript of the *ppr596* mutant, with unspliced *nad2* intron 3 highlighted by red box.

RT-PCR analysis of transcripts from mitochondrial protein-coding genes revealed a significant reduction in the accumulation of mature *nad2* transcripts in the *ppr596* mutant (Figure 3). The *nad2* transcript contains three *cis* introns and one *trans* intron (Figure 4A), and the maturation of *nad2* mRNA requires the proper splicing of these introns. To address whether the loss of PPR596 affects the splicing of these introns, we performed RT-PCR analysis in the *ppr596*, WT, and complemented plants using the primers listed in Table S1. The primer positions are shown in Figure 4A. RT-PCR analysis indicated a reduction in the splicing efficiency of *nad2* intron 3 in *ppr596* compared to the WT and complementation lines (Figure 4B). Defective splicing of *nad2* intron 3 was confirmed by the accumulation of unspliced intron 3 in the mutant plants (Figure 4C).

**Figure 5:**
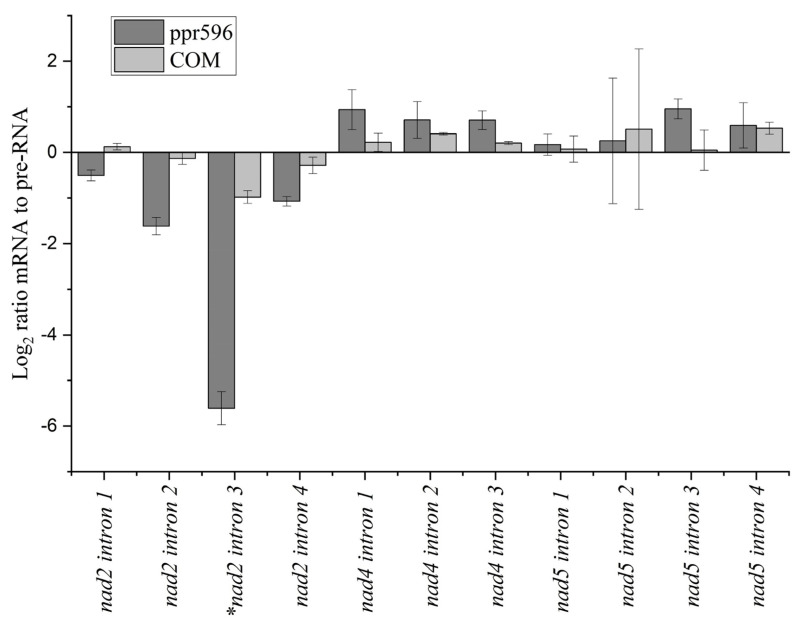
Confirmation of *nad2* intron 3 splicing efficiency through quantitative RT-PCR. The *nad4* and *nad5* introns’ splicing was used as control. The relative accumulation of mRNA and pre-RNA transcripts was analyzed in both WT and *ppr596* mutant plants. The histogram shows the splicing efficiencies, as indicated by log_2_ ratios of pre-RNA to mature RNA abundance in the *ppr596* mutant compared to the WT plants. Asterisks indicate altered splicing of *nad2* intron 3.

To verify this result by an independent approach, we conducted quantitative RT-PCR using the splicing efficiency of the *nad4* and *nad5* introns as controls. qRT-PCR results showed a (5.6-fold) decrease in the splicing efficiency of *nad2* intron 3 in mutant plants compared with the wild-type (Figure 5).

### 2.5. Biogenesis of Respiratory Complex I Was Affected in ppr596

**Figure 6:**
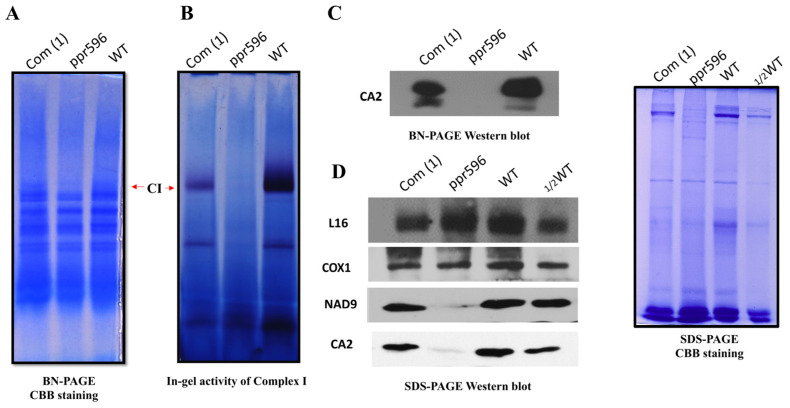
The loss of function of PPR596 affects the assembly of mitochondrial complex I. Mitochondrial proteins were isolated from 14-day seedlings of WT and complemented line, as well as one-month-old seedlings of *ppr596*, all grown in the dark. The proteins were solubilized with n-Dodecyl β-D-maltoside (β-DM), and mitochondrial membrane complexes were separated via BN-PAGE. (**A**) Coomassie Brilliant Blue (CCB) staining following electrophoresis confirms equal protein loading. The band representing complex I was reduced in the BN gel of the *ppr596* mutant. (**B**) The in-gel activity assay of complex I indicated the severe decrease in complex I in the *ppr596* mutant, with partial complementation observed in the complemented line. (**C**) Immunoblot analysis of BN-page using primary antibody for CA2, an early assembly factor of complex I. (**D**) Mitochondrion-encoded proteins L16 and COX1 were detected in WT, *ppr596* mutant, and complementation line. Crude total mitochondrial proteins from WT, *ppr596*, and complementation line were separated by SDS page followed by transformation to the polyvinylidene difluoride (PVDF) membrane. Immunoblotting analyses used primary antibodies for L16, COX1, and CA2. Coomassie Brilliant Blue (CBB) stained gels were used as the sample loading control.

*nad2* encodes a subunit of the mitochondrial complex I (CI) in the electron transfer chain. Nad2 is the core CI subunit located in the matrix arm [38]. To investigate the effect of defective splicing on the assembly of complex I, we analyzed the mitochondrial complex profile by blue native polyacrylamide gel electrophoresis (BN-PAGE), in-gel activity assay, and immunoblotting. Mitochondria were isolated from *ppr596*, WT, and complementation line seedlings grown in the dark as described [39]. Mitochondrial membrane complexes were solubilized with n-dodecyl-β-D-maltoside (β-DM), separated by BN-PAGE, and subjected to in-gel complex activity assay [40] and immunoblotting assays. Coomassie Brilliant Blue (CBB) staining of the gels revealed a significant reduction in CI in the *ppr596* mutant compared to WT and complemented plants (Figure 6A). The in-gel activity of CI indicated that the CI activity was severely reduced in *ppr596* and partially restored in the complementation line (Figure 6B). The reduction in CI assembly was further confirmed by immunoblotting using antibodies against the CI carbonic anhydrase-like subunit 2 (CA2), one of the first subunits to be incorporated in CI (Figure 6C) [41]. These results strongly support the crucial role of PPR596 in the assembly of CI.

To address the effect of reduced partial C-to-U editing of *rps3* and defective *nad2* splicing on the abundance of mitochondrial proteins, immunoblotting was performed using antibodies against L16, COX1, NAD9, and CA2. The abundance of mitochondria-encoded proteins L16 and COX1 was normal in PPR596 mutants. However, in agreement with the dramatic reduction in CI, the accumulation of CA2 and NAD9 was severely decreased in the *ppr596* mutant (Figure 6D).

**Figure 7:**
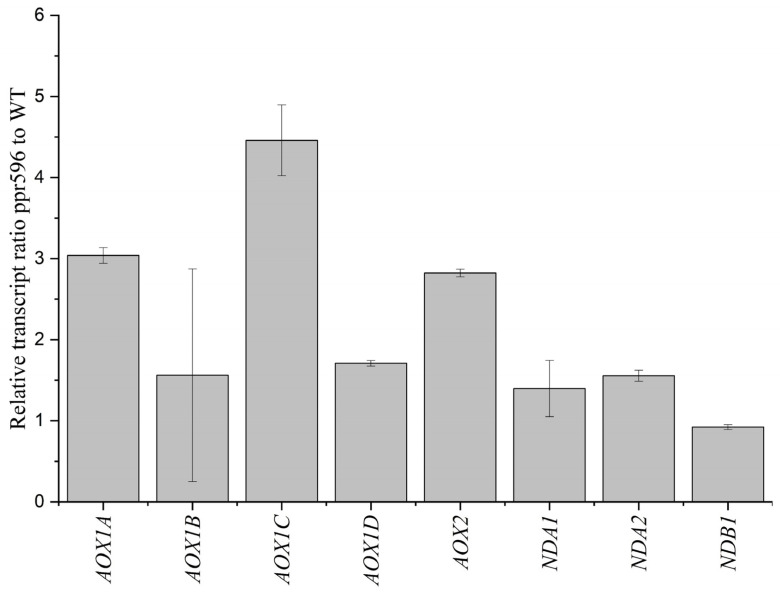
The alternative respiratory pathway is induced in the pp596 mutant. Quantitative RT-PCR results show the relative accumulation of alternative oxidase (AOX) and NADH dehydrogenase (NDA1, NDA2, NDB1) transcript in *ppr596* mutant plants.

It has been reported that the dysfunction of mitochondrial CI activates the alternative respiratory pathway as an adaptive response to mitochondrial stress [42,43,44]. To further assess the induction of alternative respiratory pathways, we measured the expression levels of various alternative oxidase (AOX) genes and rotenone-insensitive NAD(P)H dehydrogenase (NDs) in mutant plants. qRT-PCR analysis indicated that the relative accumulation of these genes transcripts was higher in mutant plants than in WT plants (Figure 7).

### 2.6. Expression Pattern and Conservation of PPR596 in Angiosperms

*PPR596* expression was evaluated by using the publicly available database The Arabidopsis Information Resource (TAIR), which indicates that the *PPR596* gene (AT1G80270) is widely expressed throughout various developmental stages of *Arabidopsis thaliana*. Its expression is notably prominent during seed germination in apical root tissues, young developing leaves, certain flower stages (particularly in carpels), and the shoot apex (Figure S3). To gain insights into its evolutionary relationship, homologous proteins of PPR596 were identified from the UniProt database using the Basic Local Alignment Search Tool (BLAST), and a phylogenetic tree was constructed. The analysis revealed that PPR596 is closely related to putative orthologs in dicots, especially with *Brassica napus* and *Nicotiana tabacum*. It formed a distinct cluster of monocots (*Triticum aestivum*, *Hordeum vulgare*, *Oryza sativa*, and *Zea mays*), suggesting that PPR596 homologs in these species may have evolved from a common ancestor (Supplemental Figure S4A). Detailed sequence alignment indicated that PPR596 is highly conserved among angiosperms (Figure S4B).

## 3. Discussion

Mitochondrial biogenesis relies on the correct expression of the mitochondrial genome. Before translation, mitochondrial transcripts undergo RNA maturation, including RNA C-to-U editing, *cis*- and *trans*-splicing of introns, and maturation of 3′- and 5′-ends. Most introns in mitochondrial transcripts are group II and require nucleus-encoded splicing factors such as maturases, chloroplast RNA splicing and ribosome maturation (CRM) domain-containing proteins, and PPR proteins [12]. PPR proteins are RNA-binding proteins involved in organellar RNA maturation events such as RNA stabilization and splicing. The P-type of the PPR protein family has predominantly been reported for its role in intron splicing, except for PPR596, PPME, and POCO1, which were reported to function in the C-to-U RNA editing [29,33,34]. PPR596 is a previously identified P-type PPR protein linked to reduced C-to-U-editing of mitochondrial *rps3* transcripts [29]. However, whether PPR596 is directly or indirectly involved in C-to-U editing is not addressed. We suspect the slight reduction in the C-to-U editing at the rps3eU1344SS site of the *rps3* transcript may not be the primary cause for the severely slow growth phenotype in *ppr596.*

This study demonstrates that PPR596 is required for the splicing of *nad2* intron 3, which is essential to the expression of Nad2 protein, a core subunit of mitochondrial CI. qRT-PCR analysis showed that the splicing efficiency of *nad2* intron 3 was severely decreased in the *ppr596* mutant, while other *nad2* transcript introns also displayed slightly reduced splicing efficiency (Figure 5). Conventional RT-PCR analysis further confirmed the severe decrease in the splicing efficiency of *nad2* intron 3 in the *ppr596* mutant (Figure 4). Many P-type PPR proteins, such as MISF26, MISF68, MISF74, ABO5, MISF2, and OTP43, play crucial roles in mitochondrial intron splicing [22,23,25,27]. Our findings suggest that the lack of PPR596 primarily affects the splicing of *nad2* intron 3, with the observed reduction in splicing efficiency of other *nad2* transcript introns possibly being a secondary consequence of impaired *nad2* intron 3 splicing. Previous research has demonstrated interdependence between the splicing efficiency of different introns [45], supported by the observations in *rug3*, where reduced splicing efficiency of *nad2* intron 3 affects the splicing effect of intron 2 [46]. This evidence strongly supports the requirement of PPR596 for the efficient splicing of *nad2* intron 3 in *Arabidopsis thaliana*.

The plant mitochondrial respiratory chain comprises five multi-subunit protein complexes (CI-CV). CI is a large L-shaped complex composed of two arms: a membrane arm embedded in the mitochondrial inner membrane, and a matrix arm protruding into the matrix [47]. In angiosperms, CI comprises approximately 50 subunits encoded by nuclear and mitochondrial genomes [48]. Nad2, a core subunit of CI, is incorporated into the membrane arm during the early CI assembly. A reduction in Nad2 leads to decreased CI assembly. Mutants with defective *nad2* splicing exhibit low CI abundance and reduced complex I activity [23,25,46,49,50]. Similarly, our BN-PAGE analysis and in-gel activity assays of CI revealed a severe reduction in CI abundance in the *ppr596* mutant compared to WT and complementation lines. Immunoblot analysis with anti-CA2 further confirmed the reduced CI assembly in *ppr596*. Complex I serves as the entry point for the electron transfer chain. The dysfunction of mitochondrial CI triggers the activation of alternative respiratory pathways in *Arabidopsis thaliana* [25,27,51]. Correspondingly, the expression of transcripts corresponding to various alternative oxidases (AOX) and rotenone-insensitive NAD(P)H dehydrogenase (NDs) was upregulated in *ppr596*, suggesting the induction of an alternative respiratory pathway (Figure 7). These findings strongly suggest the essential role of PPR596 in the *nad2* intron 3 splicing and biogenesis of respiratory complex I.

Defective splicing of *nad2* intron 3 and reduced complex I assembly are the primary cause of the observed slow growth phenotype in *ppr596*. Despite reduced partial C-to-U editing in the *rps3* transcript, *ppr596* can produce normal proteins, as in WT plants [29]. The normal accumulation of mitochondrion-encoded proteins, COX1 and L16, in the *ppr596* mutant mitochondria suggests that the partially reduced C-to-U editing in *rps3* transcripts does not strongly affect mitochondrial translation. Arabidopsis mutants defective in *nad2* splicing and complex I assembly showed similar slow growth phenotypes [23,46,49]. This evidence suggests that the primary function of PPR596 is in the efficient splicing of *nad2* intron 3, and a lack of function leads to reduced complex I assembly, resulting in the slow growth phenotype.

PPR proteins function as RNA-binding proteins by recognizing specific sequences in target RNA. The PPR code, defined by combining the fifth and last amino acids in each PPR motif, facilitates nucleotide recognition [52,53]. However, the code prediction of PPR 596 failed using the PPRCODE web server (Figure S1B) [19]. The difficulty in determining the sequence specificity of PPR proteins may be attributed to their other distinctive features [54]. The precise mechanism through which PPR596 is involved in C-to-U editing and *nad2* intron 3 splicing remains unknown. To date, two PPR proteins, ABO5 [22] and MISF26 [23], along with several other splicing factors like ABO6, CFM9, mCSF1, RUG3, mTERF15 and Organelle Zinc (OZ2) [46,50,55,56,57,58], have been implicated in the splicing of *nad2* intron 3. Exploring the interaction between PPR596 and these splicing factors could provide valuable insights into the complexities of the plant mitochondrial spliceosome.

## 4. Materials and Methods

### 4.1. Plant Material and Growth Conditions

Arabidopsis *ppr596* mutant (SAIL_367_A06) seeds were obtained from the Arabidopsis Biological Resource Center (ABRC, Columbus, OH, USA) in the Col-0 background. Before sowing, the seeds were surface sterilized with 75% ethanol with 0.05% Triton X-100 solution for 10 min, followed by 3 min sterilization with 3% sodium hypochlorite (NaClO) solution. Immediately thereafter, the seeds were washed five times with sterile water. The seeds were planted on Murashige and Skoog (MS) medium (pH 5.7) with 3% sucrose and 0.8% agar, incubated at 4 °C for three days, and then grown under long-day conditions (16 h light/8 h dark photoperiod) at 22 °C in a growth chamber. After germination on MS medium for 2 weeks, the plants were transferred to soil.

### 4.2. Plants Genotyping

DNA was extracted from green leaves using the Edwards DNA extraction method [59]. The T-DNA insertion position in the *ppr596* mutant (SAIL_367_A06) and heterozygous mutants was confirmed by PCR using the primer sets listed in Table S1.

### 4.3. Plant Complementation

The coding sequence of PPR596 was amplified by PCR from the cDNA of wild-type Arabidopsis using the primers listed in Table S1. The PCR product was successfully cloned into pENTR/D-TOPO (Invitrogen, Carlsbad, CA, USA), confirmed by Sanger sequencing, and transferred into the binary vector pGWB11 through Gateway site-specific recombination. Subsequently, the construct was introduced into *ppr596* homozygous mutant plants via *Agrobacterium tumefaciens*-mediated transformation using the floral dip method [60].

### 4.4. RNA Extraction RT-PCR and qRT-PCR

Total RNA was extracted from two-week-old seedlings of the WT, complemented lines, and one-month-old seedlings of the mutant plants using RNeasy Plant Mini Kit (Vazyme Biotech, Nanjing, China). RNA was treated with RNase-free DNase I to remove potential DNA contamination (New England Biolabs, Rowley, MA, USA). cDNA was synthesized using the Transcript FirstStrand cDNA Synthesis SuperMix (TransGen Biotech, Beijing, China). Quantitative real-time polymerase chain reaction (qRT-PCR) was performed using a LightCycler 96 (Roche, Basel, Switzerland) with three biological replicates. The Arabidopsis actin gene *AtActin* was utilized as a reference. For the functional analysis of *PPR596*, RT-PCR and qRT-PCR were performed using the primers listed in Table S1.

### 4.5. Mitochondria Isolation

Crude mitochondria were isolated from two-week-old seedlings of WT, complemented lines, and one-month-old seedlings of mutant plants grown in the dark on Murashige and Skoog (MS) medium with 3% sucrose and 0.8% agar, using a previously described protocol [39]. Fresh seedlings of the samples were homogenized with the help of a pestle and mortar on ice in extraction buffer containing 0.3 M sucrose, 5 mM tetrasodium pyrophosphate, 10 mM KH2PO4, pH 7.5, 2 mM EDTA, 1% (*w*/*v*) polyvinylpyrrolidone 40, 1% (*w*/*v*) BSA, 5 mM cysteine, and 20 mM ascorbic acid.

The homogenate was centrifuged at 3000× *g* for 5 min at 4 °C, and the resulting supernatant was centrifuged at 18,000× *g* for 30 min at the same temperature. The pellets were resuspended in a wash buffer consisting of 0.3 M sucrose, 1 mM EGTA, 10 mM MOPS-KOH, and pH 7.2. Protein concentration was determined using the Bradford method [61].

### 4.6. SDS-PAGE

The Mini-Protean system (Bio-Rad, Hercules, CA, USA) was used to perform sodium dodecyl sulfate–polyacrylamide gel electrophoresis (SDS-PAGE) on 12% Tris-HCl gels, as described previously [62], with Coomassie Blue Staining [63].

### 4.7. BN-PAGE

Mitochondrial proteins were solubilized using β-DM and separated by blue native polyacrylamide gel electrophoresis (BN-PAGE) [64]. BN-PAGE was performed at 4 °C in a vertical apparatus using blue cathode buffer (0.02% Coomassie Blue G-250) as the running buffer. Separation gels were created using linear gradients of 3–12% or 4–16% polyacrylamide (Invitrogen, Carlsbad, CA, USA).

### 4.8. In-Gel Staining

The in-gel complex activity assay was performed as previously described [40]. The complex I assay used 100 mM Tris-HCl, pH 7.5, 768 mM glycine, 0.1 mM NADH, and 0.04% nitrotetrazolium blue (NTB) (*w*/*v*).

### 4.9. Immunoblotting

The assembly of complex I was detected by immunoblotting. BN-PAGE gels were first treated with a denaturation buffer (1% SDS, 50 mM Tris-HCl, and 0.05% β-mercaptoethanol) for 30 min. For the immunoblotting analysis, mitochondrial proteins were transferred onto PVDF membranes (0.45 mm; Millipore, Burlington, MA, USA). The PVDF membranes were incubated with CA2 primary antibody against CA2, an early assembly factor of complex I as described [65]. For the immunodetection of mitochondrial-encoded proteins, proteins from the SDS page were transferred onto PVDF membranes (0.45 mm; Millipore, Burlington, MA, USA). The PVDF membranes were incubated with the primary antibodies L16, COX1, and CA2. Signal detection was carried out by ECL reagents (Thermo Fisher Scientific, Waltham, MA, USA) after incubation with the horseradish peroxidase (HRP)-conjugated secondary antibody.

### 4.10. Phylogenetic Analysis

Homologous protein sequences of PPR596 were retrieved from the UniProt database using the Basic Local Alignment Search Tool (BLAST). Alignment of these sequences from various plant species was conducted using Geneious 9.2.2 software. A phylogenetic tree was constructed using the Maximum Likelihood method and JTT matrix-based model in MEGA11. The analysis was performed using 1000 replicates [66].

## Data Availability

Data are contained within the article and supplementary materials.

## Supplementary Materials

The supporting information can be downloaded at: https://www.mdpi.com/article/10.3390/ijms25063542/s1.
