# Supplementary material for: PPR596 Is Required for nad2 Intron Splicing and Complex I Biogenesis in Arabidopsis
Source: Int J Mol Sci. 2024 Mar 21;25(6):3542. doi: 10.3390/ijms25063542 (PMC10971677; doi:10.3390/ijms25063542)
Supplement: Supplementary file 1 [file ijms-25-03542-s001.zip › ijms-2905211-supplementary figures.pdf]

PPR596 (At1g80270)

A

MPP/ICP55 cleavage site

MFALSKVLRRTQRLRLGACSAVFSKDIQLGGERSFDSNSIASTKREAVPRFYEISSLSNRALSSSAGTKSDQEEDDLED
GFSELEGSKSGQGSTSSDEDEGKLSADEEEEEELDLIETDVSRKTVEKKQSELFKTIVSAPGLSIGSALDKWVEEGNEI
TRVEIAKAMLQLRRRRMYGRALQMSEWLEANKKIEMTERDYASRLDLTVKIRGLEKGEACMQKIPKSFKG|EVLYRTLL
ANCVAAGNVKKSELVFNKMKDLGFPLS|GFTCDQMLLLHKRIDRKKIADVLLLMEKENIKPS|LLTYKILIDVKGATND
ISGMEQILETMKDEGVELD|FQTQALTARHYSGAGLKDKAEKVLKEMEGESLEAN|RRAFKDLLSIYASLGREDEVKRI
WKICESKPY|FEESLAAIQAFGKLNKVQEAEAIFEKIVKMDRRAS|SSTYSVLLRVYVDHKMLSKGKDLVKRMAESGCR
IE|ATTWDALIKLYVEAGEVEKADSLLDKASKQSHTKL|M|MNSFMYIMDEYSKRGDVHNTEKIFLKMREAGYTSR|LR
QFQALMQAYINAKSPAYGMRDRLKADNIFPNKSMAAQLAQGDPFKKTAISDILD

B

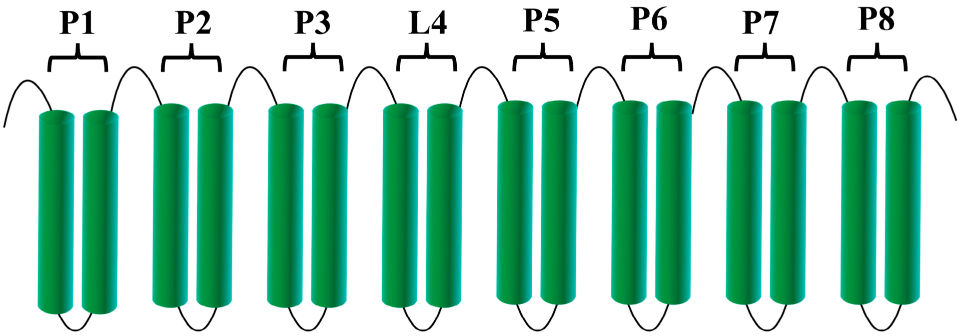

Motifs	P1	P2	P3	L4	P5	P6	P7	P8
**5th AA**	R	K	A	K	L	S	D	M
**Last AA**	S	D	N	Y	S	E	L	R
PPR code	RS	KD	AN	KY	LS	SE	DL	MR
RNA base	-	-	A>>U	?	_	_	?	?

Figure S1. Topology and binding site prediction of PPR596 protein. (**A**) The amino acid sequence of the PPR596 protein (At1g80270) is shown. Mitochondrial-targeting peptides are underlined and highlighted in red, with the cleavage site of the mitochondrial-targeted peptide indicated by a red arrow. The PPR motifs (in blue boxes) were predicted using the method described by Yan et al. (2019). Underlined letters within each motif indicate the 5th and 35th positions. (**B**) Predicted PPR codes were used to determine the RNA binding site of PPR596 protein. These results suggest that the PPR codes of PPR596 are not conserved.

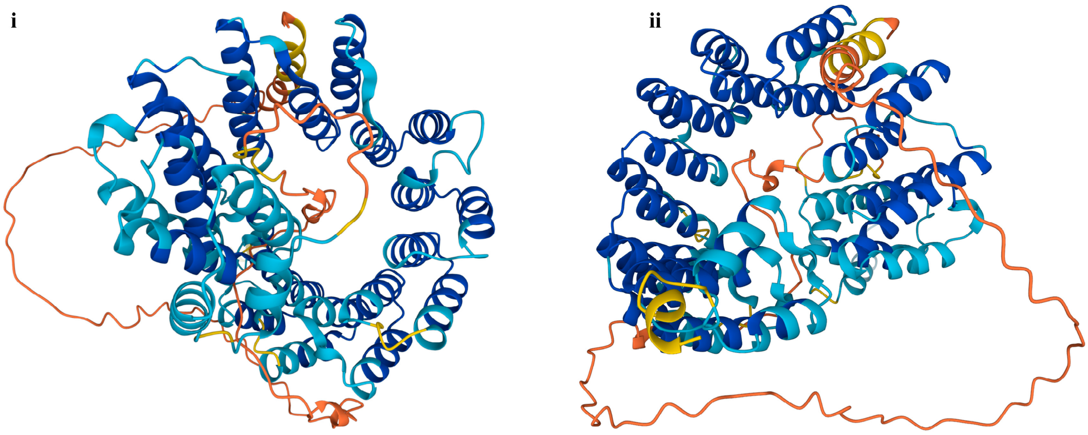

Figure S2. PPR596 protein structure. The ribbon structures (i and ii) of PPR596 were generated using the Alfa Fold server.

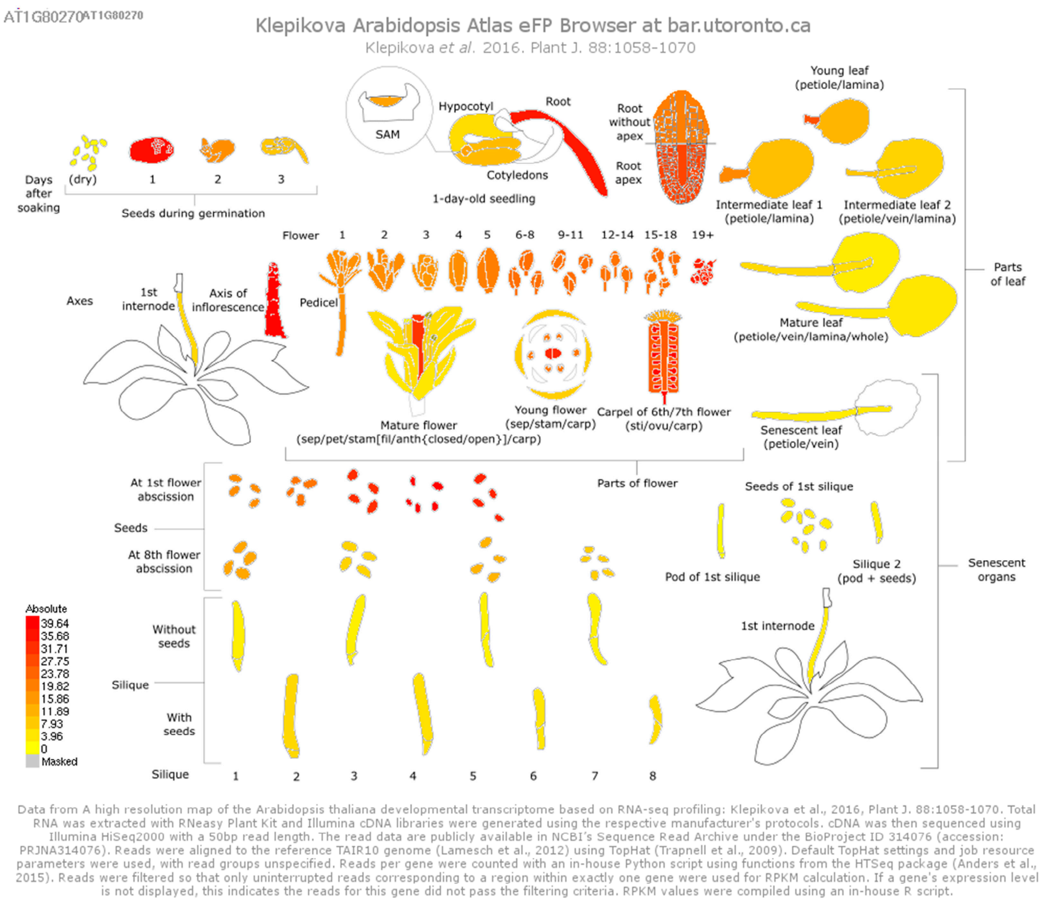

Figure S3. Expression pattern of PPR596 during *Arabidopsis* development. The expression pattern of PPR596 was analyzed using the publicly available database 'The Arabidopsis Information Resource' (TAIR; http://www.arabidopsis.org).

A

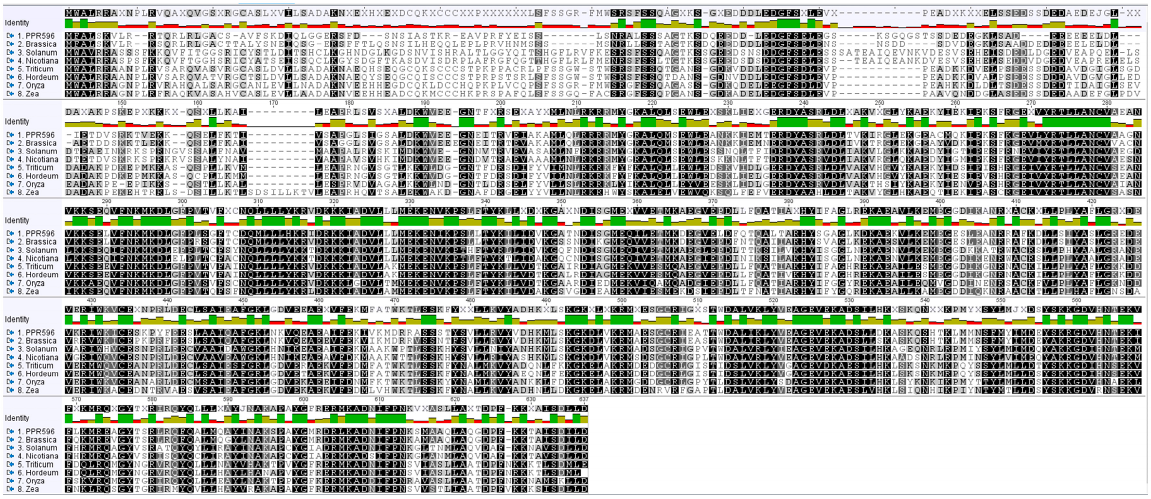

B

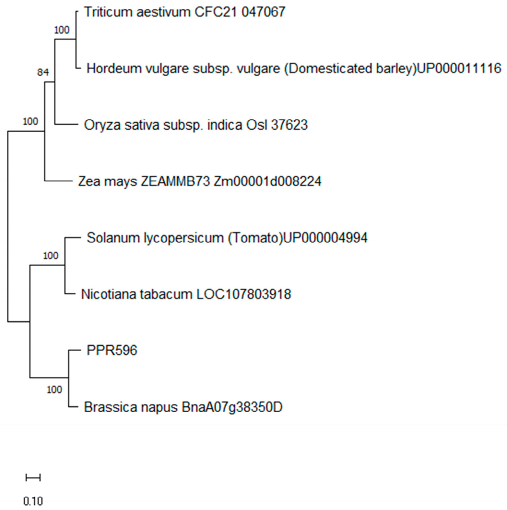

Figure S4. Phylogenetic analysis of PPR596 homologs in plants. Homologous protein sequences of PPR596 were retrieved from the UniProt database using the Basic Local Alignment Search Tool (BLAST). (**A**) An Alignment of homologous PPR596 proteins from various plant species, including *Arabidopsis thaliana* (At1g80270), *Brassica napus* (BnaA07g38350D), Nicotiana *tabacum* (LOC107803918), *Oryza sativa* subsp. *indica* (OsI_37623), *Zea mays* (ZEAMMB73_Zm00001d008224), *Triticum aestivum* (CFC21_047067), *Hordeum vulgare* subsp. vulgare (UP000011116), and *Solanum lycopersicum* (UP000004994), was constructed with Geneious 9.2.2 software. Conserved residues are shaded in the dark. (**B**) A phylogenetic tree was constructed using MEGA11 with the Maximum Likelihood method and JTT matrix-based model. The analysis was performed using 1000 replicates.
